# Supplementary material for: Branching morphogenesis in the developing kidney is not impacted by nephron formation or integration
Source: eLife. 2018 Jul 31;7:e38992. doi: 10.7554/eLife.38992 (PMC6115188; doi:10.7554/eLife.38992)
Supplement: Transparent reporting form [file elife-38992-transrepform.docx]

***eLife’s* transparent reporting form**

We encourage authors to provide detailed information *within their submission* to facilitate the interpretation and replication of experiments. Authors can upload supporting documentation to indicate the use of appropriate reporting guidelines for health-related research (see [EQUATOR Network](http://www.equator-network.org/%20)), life science research (see the [BioSharing Information Resource](https://biosharing.org/)), or the [ARRIVE guidelines](http://www.plosbiology.org/article/info:doi/10.1371/journal.pbio.1000412) for reporting work involving animal research. Where applicable, authors should refer to any relevant reporting standards documents in this form.

If you have any questions, please consult our Journal Policies and/or contact us: [editorial@elifesciences.org](mailto:editorial@elifesciences.org).

**Sample-size estimation**

- You should state whether an appropriate sample size was computed when the study was being designed
- You should state the statistical method of sample size computation and any required assumptions
- If no explicit power analysis was used, you should describe how you decided what sample (replicate) size (number) to use

Please outline where this information can be found within the submission (e.g., sections or figure legends), or explain why this information doesn’t apply to your submission:

This is primarily a descriptive study that represents the normal state in samples of different ages and not a report of differences between two different treatment groups or genotypes, so no specific sample-size estimation method was used. In order to provide a sufficient descriptive sample size of kidneys we assessed between 3-10 biological samples except for a single case where two samples at E18.5 were counted to determine tip number. In this instance the E18.5 tip numbers fit a tip curve excluding E18.5, so were therefore considered acceptable. When assessing proliferation, nephron number, and branching generation, n = 50 tips were assessed for the stated ages.

**Replicates**

- You should report how often each experiment was performed
- You should include a definition of biological versus technical replication
- The data obtained should be provided and sufficient information should be provided to indicate the number of independent biological and/or technical replicates
- If you encountered any outliers, you should describe how these were handled
- Criteria for exclusion/inclusion of data should be clearly stated
- High-throughput sequence data should be uploaded before submission, with a private link for reviewers provided (these are available from both GEO and ArrayExpress)

Please outline where this information can be found within the submission (e.g., sections or figure legends), or explain why this information doesn’t apply to your submission:

This information does not apply to our study. All analyses were on whole organs from individual animals.

**Statistical reporting**

- Statistical analysis methods should be described and justified
- Raw data should be presented in figures whenever informative to do so (typically when N per group is less than 10)
- For each experiment, you should identify the statistical tests used, exact values of N, definitions of center, methods of multiple test correction, and dispersion and precision measures (e.g., mean, median, SD, SEM, confidence intervals; and, for the major substantive results, a measure of effect size (e.g., Pearson's r, Cohen's d)
- Report exact p-values wherever possible alongside the summary statistics and 95% confidence intervals. These should be reported for all key questions and not only when the p-value is less than 0.05.

Please outline where this information can be found within the submission (e.g., sections or figure legends), or explain why this information doesn’t apply to your submission:

The statistical testing information can be found in the results section and figure legends of the manuscript, the tests used and correlative effect size (Pearson’s r) where used. We used Pearson’s r in order to identify any potential correlations, and one-way ANOVA for multi-sample tests of proliferation. One-way ANOVA tested for any potential differences between any associations of any attached or associated nephron number against tip epithelial/cap mesenchymal cellular proliferation. To do this, we treated nephron attachment and association as a factor, and the cap or tip cellular proliferation as the response variable in the ANOVA. This provided the greatest capability to denote any potential differences between any two different cases. To avoid any assumption of homogeneous variance, a Welch approximation (sometimes called Welch ANOVA) was used to determine the ANOVA which does not require normal variable data.

Details on the statistics used and the R functions used to test them available as an excel spreadsheet.

(For large datasets, or papers with a very large number of statistical tests, you may upload a single table file with tests, Ns, etc., with reference to sections in the manuscript.)

**Group allocation**

- Indicate how samples were allocated into experimental groups (in the case of clinical studies, please specify allocation to treatment method); if randomization was used, please also state if restricted randomization was applied
- Indicate if masking was used during group allocation, data collection and/or data analysis

Please outline where this information can be found within the submission (e.g., sections or figure legends), or explain why this information doesn’t apply to your submission:

This information does not apply to this study because it does not compare distinct experimental groups.

**Additional data files (“source data”)**

- We encourage you to upload relevant additional data files, such as numerical data that are represented as a graph in a figure, or as a summary table
- Where provided, these should be in the most useful format, and they can be uploaded as “Source data” files linked to a main figure or table
- Include model definition files including the full list of parameters used
- Include code used for data analysis (e.g., R, MatLab)
- Avoid stating that data files are “available upon request”

Please indicate the figures or tables for which source data files have been provided:

Fig 1A, Fig 2D, Fig 3A, Fig 3B.

All data for the quantification of branching, tip number, cell proliferation, nephron number are provided in an excel spreadsheet.
